# The Effect of 3D Printing Metal Materials on Osteoporosis Treatment

**DOI:** 10.1155/2021/9972867

**Published:** 2021-06-17

**Authors:** Bing Wang, Chuwen Feng, Jianyu Pan, Shuoyan Zhou, Zhongren Sun, Yuming Shao, Yuanyuan Qu, Shengyong Bao, Yang Li, Tiansong Yang

**Affiliations:** ^1^Heilongjiang University of Chinese Medicine, 24 Heping Road, Xiangfang District, Harbin 8615-0040, China; ^2^First Affiliated Hospital, Heilongjiang University of Chinese Medicine, 26 Heping Road, Xiangfang District, Harbin 8615-0040, China; ^3^Shenzhen People's Hospital, Second Clinical Medical College of Jinan University, Department of Rehabilitation Medicine, Shenzhen 518120, China

## Abstract

3D printing has been in use for a long time and has continued to contribute to breakthroughs in the fields of clinical, physical, and rehabilitation medicine. In order to evaluate the role of 3D printing technology in treating spinal disorders, this paper presents a systematic review of the relevant literature. 3D printing is described in terms of its adjunctive function in various stages of spinal surgery and assistance in osteoporosis treatment. A review of metal 3D printed materials and applications of the technology is also provided.

## 1. Background

3D printing technology works by applying computer design software to rapidly collect bondable materials and print them layer by layer in order to create a solid 3D model. 3D printing technology has improved dramatically since its launch in the 1990s. Although originally used in industry, the idea of “Bio-Manufacturing” with 3D printing was introduced in the late 1990s, and the technology started to be applied in the medical industry [1]. Because of its high accuracy and complexity, it has been used in orthopedic clinics to develop solid bone models, auxiliary materials for orthopedic surgery, and bone tissue replacements (implants) [2]. The spine is a complex but vital structure in the human body, with a variety of morphologies. Thus, doctors have used 3D printing technology to build spine models, intravertebral implants, guidance templates, and rehabilitation supports to increase the precision and recovery rate of spine surgery.

## 2. Application of 3D Printing Technology in Spinal Surgery

### 2.1. Basic Teaching

Any surgeon in the field of spine surgery needs to have a strong background in anatomy. Medical education is being aided by 3D printed spine models. 3D printed spine fracture models can help physicians identify complex spine fracture markers, practice prior to spine surgery, and develop standardized training programs [3, 4]. Plaster molds or specially treated cadaveric bones are the most common models for teaching spinal anatomy. However, plaster molds are not very accurate and are single models, and cadaveric bones are not always easily available and are vulnerable to ethical or legal issues [5]. Zhou et al. [6] randomly assigned 62 orthopedic residents to one of two groups. The control group learned about scoliosis using conventional methods, while the experimental group received additional training using 3D printed models. The experimental group was more successful than the control group in assessing the parietal vertebrae in scoliosis patients, as well as in identifying the structures that needed to be surgically repaired and fused (*P* < 0.05; Tables 1 and 2). The experimental group also scored higher than the control group on interest in learning, learning efficiency, interactions with the teacher (*P* < 0.01), and teamwork ability (*P* < 0.05) measures.

By using 3D printing technology to create realistic models of the spine and the aid of X-ray, CT, MRI, and other images, we can vividly describe clinical spine diseases, investigate disease causes, and discuss and practice the operative phase of surgery. However, 3D printed models have some limitations. For example, they cannot be used in basic education on a wide scale. Abstract theoretical expertise is combined with clinical cases to help train young physicians and students in surgical procedures. 3D printed models are costly and do not lend themselves well to batch manufacturing [7]. The difference between the tactile sensation of 3D printed materials and human anatomical structures can cause errors and adverse effects for practitioners as they perform surgery [8]. Moreover, 3D printed models can only reconstruct bony structures, not the surrounding soft tissues, which is not conducive to teaching precise operative techniques.

### 2.2. Preoperative Communication and Planning

Spine surgeries are demanding and complicated procedures that necessitate preoperative contact with patients and/or families in order to formulate surgical plans and stabilize the patient. Personalized surgical plans can be explored using 3D printing technology to precisely restore the target spine, and surgical rehearsals can be conducted on models to improve completion, minimize damage, shorten operation time, and formulate plans [9]. Physicians may use 3D printing to communicate the disease and treatment plan to patients who lack theoretical understanding. This helps patients appreciate the treatment, relieves their stress and anxiety, and makes them more cooperative. It also helps patients better understand surgical dangers and prognosis, reducing physician-patient disputes. For a comparative study of pedicle screw placement, Wu et al. [10] divided 62 patients with congenital scoliosis into two groups: a traditional intraoperative fluoroscopy group (C-arm group; 28 patients) and a preoperative expected model group (RP group; 34 patients). The RP group had an overall accuracy rate of 93.5%, whereas the C-arm group had an accuracy rate of 84.7%. The RP group also exhibited higher precision in screw positioning during preoperative preparation, shorter average operating period times, and less bleeding during surgery. Yang et al. [11] conducted a comparison between the groups of physicians who either did or did not use 3D printed spine models for preoperative planning. The group who did use models operated on 50 patients with adolescent idiopathic scoliosis (AIS), and the group who did not use models operated on 76 AIS patients. The former group had significantly lowered operative times, perioperative blood loss, and transfusions. 3D printing technology allows patients and doctors to view accurate and comprehensive bone models before surgery, which aids patient-provider coordination, as well as helping with the creation of highly accomplished, low-injury surgical plans. However, since 3D printed models lack surrounding tissue structures like blood vessels, nerves, and ligaments [9], they are not complete anatomical replications, and there is a risk that the surgical plan could deviate from the actual scenario. 3D printing technology also requires time for modeling: 3D printed models and patient-specific navigation templates can take up to days to prepare, depending on the size of the model and the machine used [9]. Thus, they are not suitable for preoperative planning in patients with critical conditions.

### 2.3. Intraoperative Assistance

3D printing technology for skeletal models, navigation prototypes, and personalized aids has proven to be extremely useful in complex spine surgeries. Phan et al. [12] applied a personalized fixation material made using 3D printing technology in a spinal L1-2 fusion surgery, and the patient showed significant improvement in cervical spine condition. However, the functional implications of custom-made 3D printed internal implants are not entirely clear at present, and further study is still needed. Navigation models are 3D printed from imaging data and are used to change the angle, depth, and location of the pedicle screw placement. This has four advantages [13]: the customized design to enhance nail placement accuracy; the navigation model helps streamline surgery and saves time; the procedure reduces radiation exposure during surgery; and there is reduced use of equipment, which reduces costs. Zhu et al. [14] divided 82 patients with lumbar spinal stenosis into two groups: a nail placement group which was supported by 3D printed navigation models (study group; 42 patients) and a traditional nail placement group (control group; 40 patients). The findings showed that study groups' operative time, bleeding, and mean number of fluoroscopic views were all lower than the control groups'. However, pain values (VAS) at three days and one month postoperation, as well as the lumbar spine function values (ODI) at one month postoperation, were higher. Sugawara et al. [15] published the findings of a trial in spine surgery using 3D printing-based pedicle screw guidance templates. With the aid of this reference template, 813 screws were intraoperatively inserted in 103 patients. Postoperative CT imaging confirmed that the screws were completely enclosed inside the pedicle, with no damage to the cortex, blood vessels, or nerves. Resection of spinal cord tumors with unclear boundaries [16] can be performed with the help of a 3D printed tumor model, which will reduce the degree of injury and help surgeons maintain maximum negative margins. After the tumor has been removed, 3D-printed vertebrae can be used to replace diseased vertebrae. 3D printing technology enhances surgical procedure safety, decreases the risk of surgical errors, improves surgical completion, and expands the range of options available during surgery. However, as previously mentioned, customized 3D models are time-consuming to build (taking anywhere from two hours to two days to complete), and personalized templates necessitate separate software designs [4], which is incompatible with the needs of patients with critical illnesses. Furthermore, according to one survey [17], few 3D navigation models have actually been used intraoperatively. Larger sample sizes, clinical follow-up, and testing of the basic advantages and drawbacks of 3D templates are still needed.

### 2.4. Postoperative Rehabilitation

3D printing can be used both preoperatively and intraoperatively. It can improve surgical completion accuracy, reduce trauma to patients during surgery, and allow for better prognosis and recovery. Spetzger and Wu each used individualized 3D printed intervertebral fusion devices in cervical spine surgery. Both studies found that the 3D printed models matched the patient's anatomy well and had good spinal stability, reducing the risk of postoperative dislocation and promoting postoperative rehabilitation efficiency [18]. After spine surgery, however, patients also require a period of functional exercise and rehabilitation. At this stage, 3D printing technology can be used to create customized tailored rehabilitation supports that are more closely suited to individualized spine curvatures, positions, and sizes in order to meet the various pathological needs of patients and perform more efficient functional recovery. 3D printed supports made of rich materials also outperform conventional plaster or plastic rehabilitation supports in terms of weight, comfort, and breathability [19]. Furthermore, 3D printed braces can be linked to monitoring systems for joint biomechanical analysis [9], postoperative recovery monitoring, functional exercise assistance, and adjustment of rehabilitation plans. However, due to price, material, and technology limitations, customized rehabilitation braces are only appropriate for a limited number of patients or those with complicated conditions and are not widely used in clinical settings.

## 3. 3D Printing Technology and Osteoporosis

Osteoporosis (OP) is a disorder in which bone mineral density, bone strength, and bone durability are all diminished, and a patients' BMD *T*-score (a screening method for osteoporosis) is less than 2.5 standard deviations [20]. Osteoporosis is often characterized by reduced bone tissue content, irregular bone structure, and increased bone fragility, increasing the risk of secondary fractures [21].

Geriatric osteoporosis is most common in people over the age of 70, and it is caused by tissue cellular hypofunction, which inhibits calcium absorption. Adolescents are particularly susceptible to idiopathic osteoporosis, which has no clear cause. Postmenopausal osteoporosis occurs primarily in women 5-10 years after menopause as a result of a decrease in calcitonin caused by a decrease in estrogen, which indirectly inhibits osteoclasts functioning, resulting in bone resorption, rather than bone formation [22, 23]. Patients' fracture risks are increased by changes in bone microarchitecture, progressive loss of bone strength, and progressive loss of bone mass [24]. Maquer et al. [25] showed that, due to differences in elastic properties, it is difficult to make similar bone trabeculae commercially. Barak et al. [26] used 3D printing to replicate the same structural bone trabeculae and developed standardized trabecular structural bone models (which employed different thicknesses of bone trabeculae based on differing segmentation thresholds) for use in postmenopausal osteoporosis care. Severe complications—including prosthetic interface displacement, loosening, and periprosthetic fractures—are amongst the biggest clinical challenges in osteoporotic arthroplasty [27, 28]. Furthermore, while titanium is the most widely used material for orthopedic implants due to its high mechanical strength and corrosion resistance, it is very stiff, which can result in stress shielding-induced osteolysis [29, 30]. 3D printed porous titanium (pTi) scaffolds can be significantly stiffened and printed to meet desired shapes and surface areas [31–33]. However, pTI implants can fail due to inadequate bone integration because they have very smooth surfaces and poor osteogenesis cellular adhesion [34, 35]. Dobson et al. [36] used micro-CT scans of 4 mm^3^ human bones using microstereolithography to build 3D printed models which could validate finite element (FE) predictions of osteophyte structures. The 3D printed models were tested with compression, and their stiffness values correlated strongly with the values the FE analysis had predicted. 3D printed models are an important technique to complement the use of FE models for assessing the mechanical properties of complex osteophytic structures. 3D printing technology can accurately simulate bone trabecular structures, which gives 3D printing an advantage over other conditions such as material aging, implant rejection, and fossil bone trabecular samples. 3D printing can also help personalize osteoporosis treatment and predict fracture risk [26]. Further, it can assist in curing osteoporosis by creating bone trabeculae which have incredible structural properties. However, the materials used to create these trabeculae still need further testing and refinement.

## 4. 3D Printing Metal Materials

**Figure 1:**
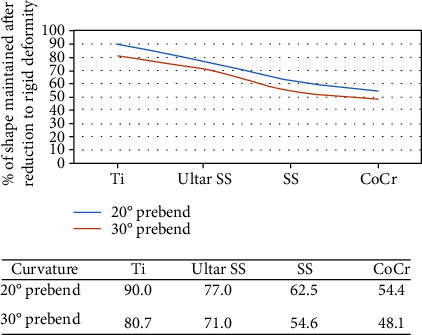
Plastic deformation of rods at 20° and 30° curvature for four materials.

Metals, ceramics, and polymers are among the numerous 3D printing materials available today. Internal implants are often used in spinal surgery, and 3D-printed internal implants are primarily made of metal and biomaterials. Stainless steel, cobalt-chromium alloy, titanium, and other metals are commonly used in clinics. Stainless steel has a lower carbon content, as well as improved mechanical and biocompatibility properties. However, it is also fragile and susceptible to low-stress values; so, it is more likely to fail under physiological loading conditions [37]. The cobalt-chromium alloy is a high-temperature alloy composed of Co and Cr with high corrosion resistance, fatigue strength, and yield strength. It has more mature applications in the medical field. However, over time, it can corrode and release harmful ions [38]. Ohrt-Nissen et al. examined stainless steel, cobalt-chromium alloy, and titanium materials that were examined in adolescent idiopathic scoliosis surgery [39]. As shown in Table 3, titanium has a high yield strength (485-1034 MPa), a low Young's modulus (110-116 GPa), and low fatigue strength (300-389 MPa). Although cobalt-chromium alloy has the highest yield strength and the lowest fatigue strength, the value span is excessive, which means there is a high possibility of instability. Stainless steel performed substantially worse than the other two types in three places. Serhan et al. conducted an experiment [40] in which 40 5.5 mm spinal rods were made from four materials: stainless steel, titanium, cobalt-chromium alloy, and ultra-high strength stainless steel (UHSS), divided equally into four groups, and then cut at 20° (*n* = 5) and 30° (*n* = 5) angles. The apical pedicle screw was subjected to a rod approximation force, and deformation of the four materials was compared. As shown in Figure 1, after being stressed under the condition of rod bending at 20°, Ti, UHSS, stainless steel, and cobalt-chromium alloy preserved 90%, 77%, 62.5%, and 54.4% of their original shapes, respectively, and preserved 80.7%, 71%, 54.6%, and 48.1% of their original shapes, respectively, after being stressed under the premise of rod bending at 30°. Thus, titanium best preserved its original morphology and had the highest overall ranking of all of the materials.

Titanium, the most widely used of these materials, has low density, high strength, low Young's modulus, high corrosion resistance, and high biocompatibility [41–43]. The most popular titanium-based materials used in traditional orthopedic endosseous implants are commercially pure titanium (CP-Ti) and Ti-6Al-4 V. However, the relatively large density, stiffness, and modulus of elasticity variations of titanium-based materials compared to human bone tissue may affect their biomedical applications [44, 45]. Therefore, adding ceramic, plastic, or other biologically inert materials to titanium-based materials can increase the yield strength (which is the stress at which permanent deformation occurs) and ultimate compressive strength. Titanium alloy, as the primary metal material used in clinical applications, has high reconstructed spine structure stability and high yield strength, but low stiffness. Furthermore, titanium alloy-based microporous metal implants have higher safety and efficacy scores. Zhang et al. [44] compared the Vickers hardness, yield strength, compressive ultimate strength, and maximum strain of composites produced by selective laser melting (SLM) to those of CP-Ti and Ti-6Al-4 V produced by other techniques.  Table 4 [46–50] shows that the Vickers hardness of Ti-TiB (402 HV) was greater than that of CP-Ti (210 HV) and Ti-6Al-4 V (346 HV); the yield strength of Ti-TiB (1103 MPa) was greater than that of CP-Ti (700 MPa) and Ti-6Al-4 V (1000 MPa), and that the compressive strength and maximum strain values of Ti-TiB are greater than those of typical CP-Ti and Ti-6Al-4 V. While differences in fabrication techniques can play a role, clinical trials show that titanium alloys are much more effective than pure titanium. Additionally, although titanium has been used in spine surgery for a long time with good results, findings are often focused on a small, individualized number of cases. It is unknown whether the mechanical strength and properties of titanium alloys would be compatible with current data if 3D printing technology could achieve mass production, and ongoing evaluation studies are needed [13]. There are fewer types of materials that lend themselves well to 3D printing, because they must simultaneously meet the complex requirements of protection, compatibility, and degradability [38]. Additionally, 3D printing materials must undergo clinical trials before they can be used in manufacturing. Materials research that involves modifying the structural shape of materials or research that involves mixing metals with biological cellular materials, could lead to more application possibilities.

## 5. Outlook

The spine is one of the body's most significant skeletal structures, and spine surgery is a meticulous operation that involves disc structures, adjacent tissues, physiological curvature, and gravity effects. 3D printing technology has only been in use for a few decades, but it has already led to breakthroughs in orthopedic spine surgery, solving issues that were previously unsalvageable and providing further hope for medical progress. 3D printing is still an emerging technology; however, relevant regulations have not yet been perfected, clinical applications are limited due to high costs, and it has not yet been applied widely outside of a few complex cases. Nevertheless, with recent emphasis on innovative medical technologies, the utility of raw materials for 3D printing has increased, the costs are decreasing, and efficacy is improving. Different 3D printing structures and materials are required when it is applied to different types or degrees of osteoporosis, but the therapeutic effects for this condition still need further research. The development of 3D printing technology in medicine also involves industrial production, software design, physical and chemical research, and many other fields. For example, raw material research may increase material potential by modifying its structures, altering manufacturing techniques, changing joining methods, and expanding the variety of materials available. In addition to metal materials, which are the most commonly used, bioprinting materials have started to receive attention in recent years. Active biomaterials with nutrients are more suitable for in vivo placement. However, further research is needed to mitigate rejection of nonautogenous cells. Other 3D printing research is related to the manufacturing of controlled-release drugs and their application to the rehabilitation phase of spinal surgery. Controlled-release drugs are introduced into internal implants using 3D printing, and the drug effect is quantitatively and directly applied to the spinal site to optimize postoperative rehabilitation. Future 3D printing research could also network platforms using artificial intelligence, collect big data, and exchange and summarize the success of research experiments, thus laying the groundwork for the standardization and popularization of 3D printing technology. Much of research on 3D printing is still in its early stages, but it is certain to have broader medical applications and benefits in the future.

## Figures and Tables

**Table 1:** Comparison of the correct rate of scoliosis tests.

Project	Experimental group (%)	Control group (%)	*χ* ^2^	*P*
The apical vertebrae of scoliosis	83.8	58.1	5.01	0.025
The planned surgery of segment	77.4	51.6	4.51	0.034

**Table 2:** Comparison of questionnaire assessment results.

Project	Experimental group (${\bar{\chi }}^{\pm s}$)	Control group (${\bar{\chi }}^{\pm s}$)	*t*	*P*
Learning interest	(4.26 ± 0.86)	(3.26 ± 1.27)	3.28	<0.01
Learning efficiency	(3.81 ± 0.83)	(3.0 ± 0.73)	4.29	<0.01
Proactive interaction	(4.1 ± 0.79)	(3.42 ± 0.77)	3.50	<0.01
Teamwork ability	(3.1 ± 1.01)	(2.65 ± 0.61)	2.19	<0.05

**Table 3:** Mechanical properties of three major spinal implants in AIS surgery.

Biomaterial	Young's modulus/elastic modulus (Gpa)	Yield strength (MPa)	Fatigue strengh (MPa)
Stainless steel	190	792	241-820
CoCr alloys	200-300	300-2000	207-950
Titanium alloys	110-116	485-1034	300-389

**Table 4:** Comparison of the Vickers hardness and compressive properties between Ti–TiB, CP-Ti, and Ti–6Al–4 V composites.

Reference	Material type	Condition	Vickers hardness (HV)	Ultimate comprehensive strength (MPa)	Yield strength (MPa)	Maximum strain (%)
[27, 28]	CP-Ti	Casting/ECAP	210	900	700	35
[29, 30]	Ti-6AI-4 V	Superplastic forming/annealed	346	1300	1000	10
[31]	Ti-TiB	SLM	402	1421	1103	17.8

## Data Availability

There is no laboratory data in this study, and the review process and references are corrected and put in the Data Center of Heilongjiang University of Chinese Medicine for 8 years.
